# Body-weight support gait training in neurological intensive care: safety, feasibility, and delays before walking with or without suspension

**DOI:** 10.1186/s12984-023-01291-9

**Published:** 2023-12-13

**Authors:** Claire Jourdan, Fanny Pradalier, Kevin Chalard, Margrit Ascher, Francisco Miron Duran, Frédérique Pavillard, Frédéric Greco, Myriam Mellouk, Stéphane Fournier, Flora Djanikian, Isabelle Laffont, Anthony Gelis, Pierre-François Perrigault

**Affiliations:** 1 Département de Médecine Physique et de Réadaptation, CHU de Montpellier, Hôpital Lapeyronie, Site Lapeyronie, 371 Avenue du Doyen Gaston Giraud, 34295 Montpellier cedex 5, France; 2 Département d’anesthésie-Réanimation Gui de Chauliac, CHU de Montpellier, Montpellier, France; 3 Euromov Digital Health in Motion, Université de Montpellier, Montpellier, France

**Keywords:** Early mobilization, Neurocritical care, Brain injury, Rehabilitation, Body weight support, Verticalization

## Abstract

**Background:**

Early Mobilization in Intensive Care Units (ICUs) enhances patients’ evolution, but has been rarely studied in neurological ICUs. The aim of this study was to assess gait training with body-weight support (BWS) in neuroICU, and to report on its safety, feasibility and on delays before walking with and without BWS.

**Methods:**

This study was an observational one-year single-center study. Inclusion criteria were adults with a neurological injury requiring mechanical ventilation. Exclusion criteria were early death or ICU transfer. After weaning from ventilation, patients were screened for indications of BWS walking using predefined criteria.

**Results:**

Patients’ conditions were mostly brain injuries: 32% subarachnoid hemorrhages, 42% focal strokes, and 12% traumatic brain injuries. Out of 272 admissions, 136 patients were excluded, 78 were eligible, and 33 performed BWS walking. Among non-eligible patients, 36 walked unsuspended upon ventilation weaning, 17 presented too severe impairments. Among the 45 eligible patients who did not receive BWS training, main reasons were workload and weekends (31%), medical barriers (29%), and early ICU discharge (22%). 78 BWS sessions were performed on the 33 beneficiaries (median sessions per patient 2, max 10). Pre-session, most patients had inadequate response to pain, orders, or simple orientation questions. Sitting without support was impossible for 74%. Most pre-post changes in hemodynamic, respiratory, and pain parameters were small, and recovered spontaneously after the session. Eight sessions were interrupted; reasons were pain, fatigue or major imbalance (4), syncope (1), occurrence of stool (2), and battery failure (1). None of these adverse events required medical intervention, patients recovered upon session interruption. Median session duration was 31 min, patients walked on median 17 m. First BWS session occurred on median 3 days after ventilation weaning, and 11 days before patients were able to walk unsuspended.

**Conclusions:**

Verticalization and walking using a suspension device in patients in neuroICU allows early gait training, despite challenging neurological impairments. It is safe and generally well tolerated.

*Trial registration*: ClinicalTrials database (ID: NCT04300491).

## Introduction

The benefits of Early Mobilization (EM) in critical care has been demonstrated on the duration of mechanical ventilation, on lengths of stay, and on functional outcomes [1]. This has made EM a common practice in Intensive Care Units (ICUs) [2]. The basic principle of EM is that patients realize motor exercises of increasing intensities along their clinical evolution—from passive in-bed mobilizations to active out-of-bed motor training. Step-by-step progress of EM is made as early as clinically feasible (eg. bedside sitting, bed-to-chair, bedside standing, walking) [2]. International recommendations [3] and National Guidelines [4] provide guidance for EM.

EM research has mostly been performed in general or surgical ICUs, and primary conditions of patients were respiratory, cardiac or septic failure [5]. These studies included few—if any—patients with primary neurological failure, although EM is presumably beneficial for critical neurological patients also [6]. Data on safety, benefits, and EM strategies for these patients is lacking. In particular, patients with critical brain injuries have clinical specificities which make EM implementation challenging [7]. The need to control the intracranial pressure and the cerebral blood flow prevents the early interruption of sedation. Consciousness and speech disorders limit patient participation. Motor, sensitive and balance deficits reduce active mobilization capacities and increase the risks of falls.

As such, verticalization is challenging in neurological ICUs without specific devices. Previous reports illustrate the use of tilt tables [8], which might integrate robotic stepping devices—the Erigo® system [9]. This passive verticalization could promote arousal for disorders of consciousness [9].

When consciousness levels allow more active training, EM should progress towards bedside standing and walking, but this is likely to be prevented by neurological impairments. Body weigh-support (BWS) systems might at this stage be used. BWS has been greatly tried in chronic stroke sequelae [10]. It may be effective in acute stroke rehabilitation [11], where it tends to result in more rapid access to independent walking [12]. Various devices exist, including suspension systems associated with treadmills, with robotic-assisted gait training, or with exoskeletons, but few of them are mobile enough to be used in an ICU unit.

The present study describes the use of a mobile body-weight support device to allow walking training soon after weaning from ventilation for patients requiring neuroICU care. Its aims were (1) to evaluate the safety of training sessions (changes in clinical parameters, occurrence of adverse events); (2) to specify the feasibility of suspended gait training in neuroICU (characteristics and proportion of eligible patients, caregivers' time and number required, reasons for missed sessions); (3) and to assess whether the use of a suspension device could shorten the delays before gait training initiation, by measuring the time-interval between BWS walking and walking without suspension.

## Material and methods

### Study center

A single-center observational study was conducted in the 16-bed neurological ICU of Montpellier University Hospital from January 2018 to end-January 2019. Patients with poor-grade subarachnoid hemorrhage, severe ischemic or hemorrhagic stroke, spinal cord injuries or isolated traumatic brain injuries are admitted in this ICU. The mean length of stay of these patients is 12 days. Almost all of them require mechanical ventilation and 25% of them die in the hospital.

The unit has gradually implemented EM for the last 10 years. All patients receive one or two rehabilitation sessions per day. Session duration depends on patient’s need and tolerance. Rehabilitation prescriptions are completed daily by intensivists and specified twice weekly during multidisciplinary meetings with Physical Medicine and Rehabilitation practitioners.

### Intervention

Since 2017, the unit realizes walking sessions with a mobile suspension device, the LiteGait® system, model LG 400, maximal weight 200 kg. This device offers total or partial body-weight support for patients, without requiring them to stand up to install its harness, and allows suspension for bedside standing as well as walking in corridors. The unit’s clinical criteria to perform a BWS walking session are: (1) hemodynamic and respiratory stability without mechanical ventilation as defined in French guidelines for EM [4]; (2) absence of raised intracranial pressure, ongoing vasospasm, uncontrolled epilepsy, or other neurologically unstable condition; (3) patient conscious and able to participate; (5) patient able to hold his head up; (6) tolerance of (passive or active) sitting position. Generally, beneficiaries are stable with medical support (oxygen, medication), participating but not fully awake, globally hypotonic and with various neurological deficiencies.

The device used and settings were identical across sessions, excepted for the harness, which offers three different sizes. All sessions were guided by one of the two physiotherapists of the unit, who have been previously trained to use the device. To install the BWS system, the harness is tightened around the patient while lying in bed. The patient is helped to transfer to bedside sitting position, and the physiotherapist fixes the harness straps to the telescopic arm of the LiteGait structure, which is then raised to lift the patient into a standing position. Medical devices (lines, oxygen, pumps…) are interrupted if possible, or attached to the device or held by caregivers. External ventricular shunts are temporarily clamped. The weight-bearing assistance is adjusted by the physiotherapist throughout the session (min 20% weight support, max 90%), to allow the patient to walk in the ICU corridors, with correct control of knee extension, while on maximal tolerated weight bearing. Duration of sessions depends on patient tolerance—they last generally half on hour, which includes 10 min of walking. At the end of the session, the patient is installed on a chair. Besides the physiotherapist, two caregivers are generally required at the start of the session (patient installation, adaptation of medical devices), and one caregiver during the walking part of the session.

### Study population

All patients with neurological injuries admitted for 48 h or more and requiring mechanical ventilation were screened. Exclusion criteria were age below 18, premature death, early discharge from the ICU before weaning from ventilation, and opposition of patient.

To select patients for body-weight support (BWS) walking, patients were systematically assessed for indications of BWS, after the weaning of ventilation. Every weekday, physiotherapists assessed all non-ventilated patients. Patients were selected by physiotherapists for BWS walking if the aforementioned criteria were present. Patients were not selected if they could walk without BWS, or if neurological impairments were too severe (eg. persistent disorders of consciousness, quadriplegia).

Among patients with indication for BWS walking, some benefited from one or more BWS sessions, and some did not receive any. For this last group, reasons for not providing BWS sessions were described (limitations related to staff constraints, to the device, or to the patient). The group of beneficiaries represented the study sample.

### Data collection

In the study sample, patient demographic and basic clinical data were retrospectively collected through medical records and ICU follow-up charts, by two research medical practitioners who were blinded to the conduct of BWS sessions. Patients’ clinical data included injury type and severity, global motor assessment upon ICU discharge (quadriplegia, hemiplegia, hemiparesis, no impairment), duration of ventilation, duration of stay in ICU and in hospital, and delay before walking without suspension.

Regarding BWS walking sessions, all the data was collected without blinding by one of the two physiotherapists of the unit, while performing the session. Data regarding BWS session was collected on standardized forms, which included pre-session and post-session clinical evaluations, and description of sessions. Pre-session assessments included pain assessed by the Behavioral Pain Scale-Non Intubated [13], consciousness assessed by the Richmond Agitation-Sedation Scale [14] and the Edinburgh-2 Coma Scale [15] (a rapid scale assessing response to orders, to pain, and to two simple orientation questions), Medical Research Council (MRC) testing of each quadriceps (0–5), sitting balance derived from the PASS scale [16] (3 levels: cannot sit, sits with support, sits without support), systolic blood pressure (BP), heart rate (HR), and oxygen saturation (SpO_2_). Post-session parameters included pain, BP, HR and SpO_2_. Description of sessions included eventual premature session interruptions, their reasons, and adverse events. It also included session duration, number of caregivers involved, walking duration, walking distance performed, and occurrence of a spontaneous smile.

### Data analysis

Characteristics of the sample were described; median and interquartile range (IQR) were used, since distribution of most quantitative variables were non-normal. Analysis of BWS walking sessions pooled all sessions performed. Descriptive statistics were presented, along with histograms of pre-post session changes for clinical parameters (BP, HR, SpO_2_, pain).

In order to represent delays before (1) weaning of ventilation, (2) first BWS walking, and (3) first unsuspended walking, survival curves using the Kaplan–Meier method were computed [17]. The curves showed the probability of reaching each successive goal at increasing times from initial intubation, goals being treated as events. The maximal delay considered was 150 days, information on walking ability and their delays was obtained by contacting post-hospital care centers when necessary. If the information on post-hospitalization walking ability was unavailable (n = 2 cases), the patient’s data was right-censored at the date of hospital discharge—the survival analysis considered these cases as lost-to-follow-up from the date of censoring, and subsequently calculated proportions of patients who reached unsuspended walking with the remaining patients. Statistical analyses were computed with R® [18], version 3.6.0; survival curves used the package Survival [19].

## Results

### Population description

**Fig. 1:**
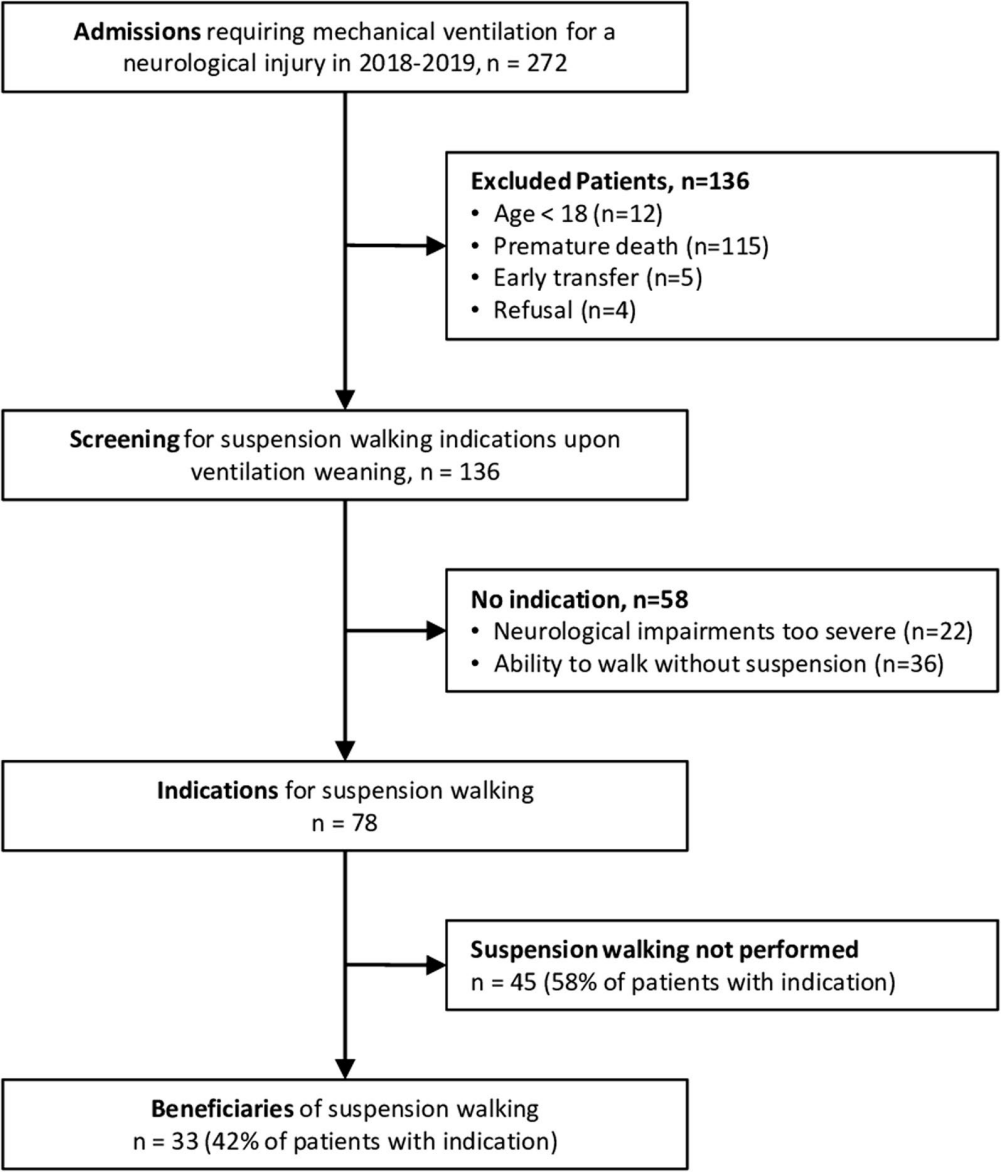
Study flow chart. *ICU* intensive care unit

Out of 272 patients with neurological injuries requiring mechanical ventilation, 33 patients received BWS walking training (see flowchart Fig. 1, and Table 1 for sample characteristics). Thirty-two patients had sustained brain injuries; 12% traumatic, 76% strokes, 9% other brain injuries. One patient had incomplete cervical spinal cord injury.

**Table 1:** Characteristics of patients who received suspension walking training (n = 33)*

	Count (%) or median (IQR)
Age (years)	54 [41–66]
Gender (male)	20 (61%)
Cause of neurological injury	
Subarachnoid hemorrhage	11 (32%)
Ischemic stroke	7 (21%)
Hemorrhagic stroke	7 (21%)
Traumatic Brain Injury	4 (12%)
Other	4 (12%)
Initial GCS score	10 [6–13]
Initial SAPS II	50 [38–55]
Initial weight (kg)	77 [69–86]
Invasive ventilation duration (days)	19 [10–28]
Tracheotomy (yes)	4 (12%)
Motor status	
Quadriparesis	4 (12%)
Hemiplegia	12 (36%)
Hemiparesis	11 (33%)
No major impairment	6 (18%)
ICU length of stay (days)	27 [21–35]
Hospital length of stay (days)	39 [33–65]

Only one value was missing for the table (weight of one patient)

*IQR* interquartile range, *GCS* Glasgow coma scale, *SAPS* simplified acute physiology score, *ICU* intensive care unit

^*^Values refer to count (percentage) or median [interquartile range]

For the 45 patients with indication for BWS training who did not receive it, reasons appear in Table 2. Delay from ventilation weaning to ICU discharge was shorter in this group (median 3 days, IQR = 2–5) than in the group of beneficiaries (median 8 days, IQR = 4–15, p < 0.0001).

**Table 2:** Reasons for not realizing body weight support (BWS) walking sessions (n = 45 patients)*

Obstacles to realizing BWS	Patient count (%)
Early ICU discharge (before BWS session)	10 (22%)
Limited staff, time constraints	
Limited staff for physiotherapy on weekends days	5 (11%)
High workload of physiotherapists	9 (20%)
Limitations related to the device	
Battery failure	3 (7%)
Patient-related barriers	13 (29%)
Fatigue	1
Femoral or foot catheter	3
Craniectomy with no protective headgear	4
Sacral skin pressure ulcer	1
Recent coronary syndrome	1
High oxygen needs	2
Surgical contraindication	1
Unknown	5 (11%)

*ICU intensive care unit

### Description of BWS walking sessions (Table 3)

**Table 3:** Characteristics of body weight support walking sessions (n = 78)

	Count (%) or median [interquartile range]	Missing data count (%)
Patient medical devices		
External ventricular drain	12 (14%)	0 (0%)
Central venous and/or arterial catheter	45 (53%)	
Femoral catheter	5 (6%)	
Electric syringe pump	12 (14%)	
Tracheostomy	7 (8%)	
Oxygen therapy	35 (41%)	
Bladder catheter	61 (72%)	
Patient pre-session clinical parameters		
Richmond agitation-sedation scale		
Drowsy (− 1)	1 (1%)	0 (0%)
Alert and calm (0)	68 (80%)	
Restless (+ 1)	6 (7%)	
Agitated (+ 2)	3 (4%)	
Edinburgh-2 Coma Scale		
Response to two orders (none/1 correct/2 correct)	8 (9%)/7 (8%)/62 (73%)	1 (1%)
Response to pain (none/inadequate/adequate)	13 (15%)/34 (40%)/30 (35%)	
Correct orientation answers (none/one/two)	40 (47%)/15 (18%)/22 (26%)	
Strength of each quadriceps (right/left)		
0 = No contraction	13 (15%)/8 (9%)	0 (0%)
1 = Contraction	1 (1%)/9 (11%)	
2 = Movement not against gravity	24 (28%)/22 (26%)	
3 = Movement against gravity	37 (44%)/34 (40%)	
4 = Movement against resistance	3 (4%)/5 (6%)	
5 = Normal strength	0 (0%)/0 (0%)	
Sitting balance		
Cannot sit	17 (20%)	4 (5%)
Sits with support	46 (54%)	
Sits without support	11 (13%)	
Description of suspension walking sessions		
Number of caregivers required		0 (0%)
One	1 (1%)	
Two	40 (47%)	
Three	32 (38%)	
Four	5 (6%)	
Median session time (minutes)	31 [26–45]	0 (0%)
Median walking time for the patient (minutes)	8 [5–10]	0 (0%)
Median walking distance (m)	17 [10–30]	0 (0%)

A total of 78 BWS sessions were performed on the 33 beneficiaries. Eight patients received one session, 14 received two, 8 received three, and three patients received respectively five, eight, and ten sessions.

Pre-session assessments showed that most patients had not regained complete consciousness on the Edinburgh scale. Most sessions were realized with one or more medical devices, predominantly central vascular catheters and bladder catheters. Sitting without support was impossible in 74% of cases. On the MRC testing, quadriceps were never at the normal 5-point strength, and very rarely (4% and 6%) at the subnormal 4-point strength. In a third of sessions only, patients had both quadriceps at 3 or more, meaning able to resist to gravity—in the majority of sessions, one or both quadriceps had a strength weaker than 3.

Half of the sessions (47%) required two caregivers (including the physiotherapist), 38% required three caregivers. Median total session time was 31 min, median time spent walking was 8 min, median walking distance was 17 m.

**Fig. 2:**
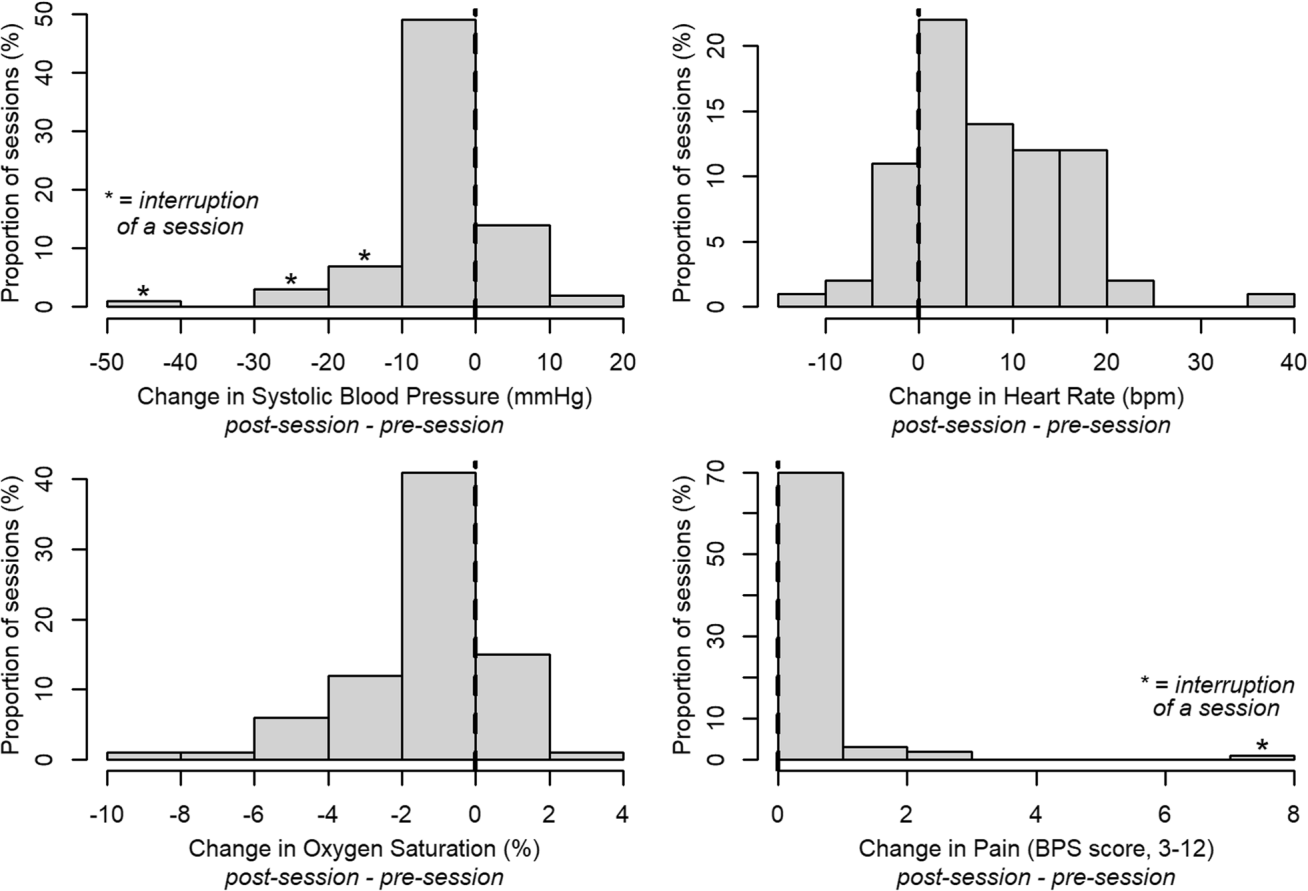
Changes in clinical parameters after the suspension walking sessions (n = 78). Graphs represent frequency histograms of differences = post-session values—pre-session values. Stars (*) indicate that one of the sessions of the bar was interrupted prematurely due to clinical intolerance. *BPS* behavioural pain scale

Most changes in hemodynamic, respiratory, and pain parameters were small, as illustrated in Fig. 2. Four patients had sBP drops of 20 mmHg or more. Maximum loss of oxygen saturation was 9. Eight sessions were interrupted prematurely, for the following reasons: battery failure (1), pain related to the device’s harness (1), occurrence of stool (2), posterior pushing (1), excessive fatigue (1), dizziness (1), and syncope (1)—the last three being accompanied by drops in sBP of 10 to 50 mmHg. Regarding the session interrupted because of a syncope, the patient recovered normal blood pressure and consciousness immediately after bed rest. No adverse event required a medical intervention or had clinical consequences other than the interruption of the session. In 43 cases (51%), the BWS walking provoked a spontaneous smile on the patient’s face.

### Delays before walking with or without BWS (see Fig. 3)

**Fig. 3:**
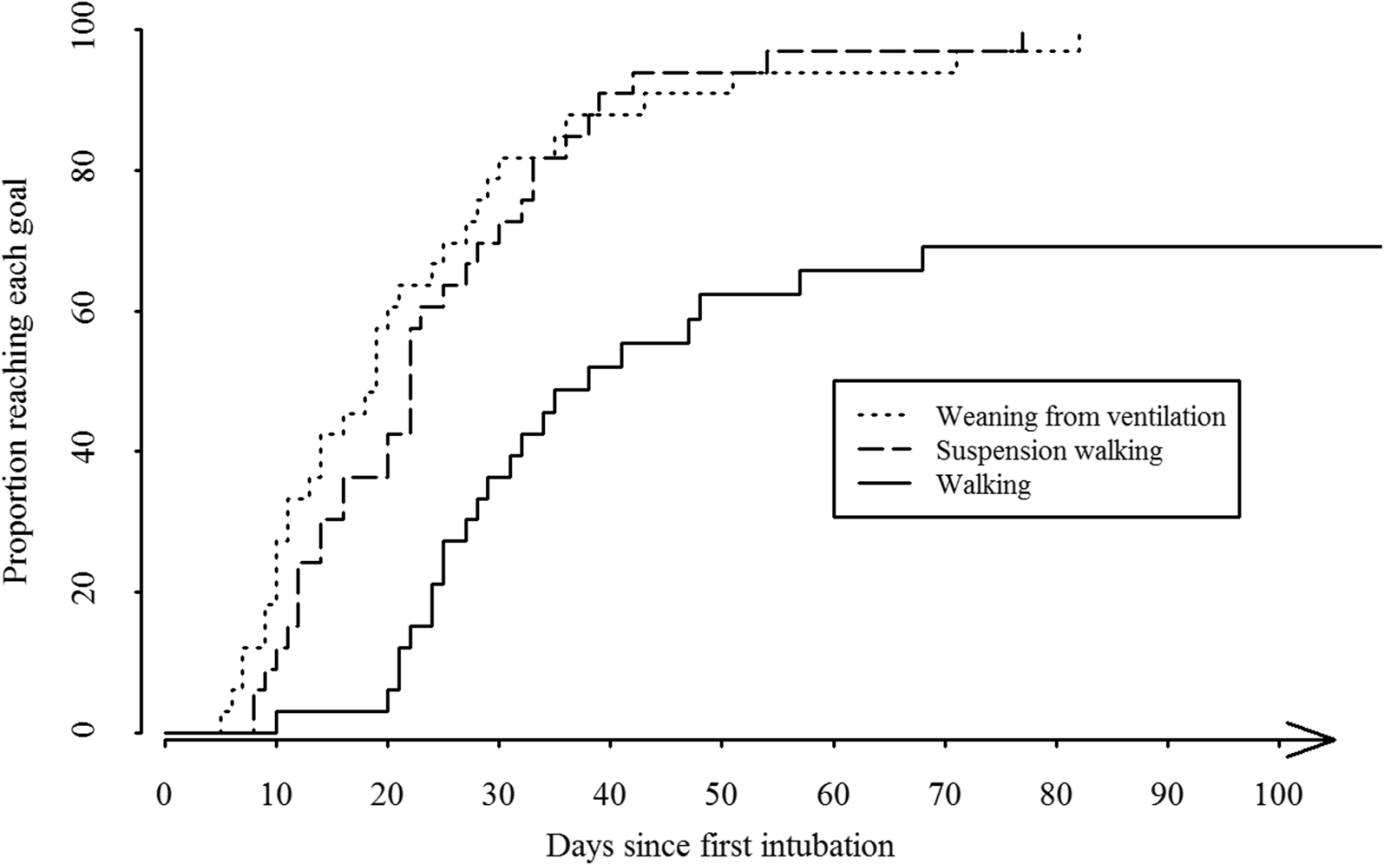
Kaplan–Meier curves for delays from intubation to (1) weaning from ventilation (2) first suspension walking session in ICU (3) first walking session without suspension, in ICU or hospital wards. Graphs represent proportion of patients in the study sample (n = 33) who reached each goal at each time-point post-intubation. *ICU* intensive care unit

First BWS session took place at a median of 3 days (IQR = 2–6) after weaning from ventilation. For patients who resumed walking without BWS during their hospital stay (n = 22), the first unsuspended walking session took place at a median of 11 days (IQR = 4–16) after initiation of walking with suspension.

## Discussion

This observational study illustrates the safety of walking for patients in neurological ICUs, even rapidly upon arousal and extubation, when using a body-weight support (BWS) device. A total of 33 patients benefited from 78 BWS walking sessions over a year, with few—and transitory—adverse events. It also showed that it is feasible and can be integrated in an early mobilization (EM) program in a neurological ICU. An important result is that patients who benefited from BWS sessions had a clinical status (incomplete arousal, lack of balance, insufficient strength) which would have prevented any bedside standing or walking without the suspension system.

The safety and tolerance of the technique, at such an early stage in the patient’s care, is remarkable. Excepted in three sessions, drops in systolic blood pressure did not exceed 20 mmHg; heart rates and oxygen saturation showed little changes. Clinical tolerance was also satisfactory in terms of neurological status, behavior, and pain: there were no problems of participation, no session interruption related to patients’ agitation. Moreover, a majority of patients smiled during sessions, suggesting they found enjoyment in walking with suspension. These findings were consistent with those of Sottile and al. [21], who showed that the perception of physiotherapy in ICU by patients was positive, and with the quantitative assessments of enjoyment during EM performed by Hickmann and al. [22], which was found to be highest when walking.

This study illustrates the need to use specific equipment for EM in ICU. Lack of equipment is a classical barrier for EM [23, 24]. In ICU reports of EM for patients with respiratory failure, walking training uses traditional walkers [25], or custom-made devices which might carry oxygen, intravenous poles, ventilators [26]. It appears here that BWS devices are a specific requirement for verticalization and walking in neurological ICUs, given patients’ neurologic impairments. BWS devices might furthermore be relevant in general ICUs, since a recent randomized trial found a trend for shorter delays before independent walking and significantly shorter lengths of hospital stay in non-neurological patients trained for walking with BWS in critical care [27].

This study also highlights the need for continuous efforts to overcome well-described barriers to EM implementation [23, 24]. A total of 45 patients with indication for BWS walking did not receive it. In 13 cases, reasons were medical barriers or safety concerns, some of them being avoidable. Need for protective headgears for craniostomies might be anticipated. EM with in situ femoral catheters was found safe in multiple studies [28, 29], and previous experience in our unit was consistently safe, but presence of lines nonetheless prevented walking training in some cases. Education of staff and EM protocols are helpful to overcome these safety concerns [24]; while most EM protocols clearly define medical contraindications, it seems critical that such protocols insist on situations which should not be regarded as limitations for EM.

In a high number of cases, organizational barriers such as heavy workload or weekends prevented BWS training. In a dedicated review on barriers to EM, Parry et al. [24] found workload and lack of staff cited by more than 15 studies. The high level of caregivers’ time required for BWS walking sessions in this study might discourage neuroICU teams to implement BWS walking. Yet other EM activities (bedside sitting, active transfer to chair) for patients with these levels of impairments and medicalization would likewise require time and implication of the whole staff, a consistent characteristic of EM [4]. The necessity of restructuring roles and responsibilities to permit EM has been stressed before [24]. Given the cost-efficacy of EM in ICU [30], and previous experiences on successful implementation of EM when adequate funding and staffing is provided [31], these results are one more advocacy to allocate staffing for EM. Besides, the capacity to make patients with severe neurological injuries walk in ICU, soon after extubation, has been a strong motivational factor for our team. Indeed, a key to the success of EM is to obtain buy-in for this time-requiring activity [23].

The methodology of the present study did not allow any conclusions to be drawn as to the benefits of BWS walking in ICU on patients’ outcome. But it showed that it accelerated steps of EM: the technique enabled the training of gait to be anticipated by a median of 11 days. Indeed, the provision of gait training very early after critical brain injuries is likely to be beneficial on outcome. In order to enhance brain plasticity in post-stroke rehabilitation [32], the priority is to provide task-oriented training, as soon as possible [33]. Higher doses of walking training in the first weeks post-stroke are associated in recent RCTs with greater walking capacity [34, 35], which are maintained over the first year post-stroke [35].

The strength of this study was that it provided real-life data on the practical use of an EM technique, with information on rates of beneficiaries, challenges and benefits, and on ways to improve EM and walking in neuroICUs. The population of the study was very specific, all but one patient had suffered from severe brain injuries, essentially strokes, and all had required invasive ventilation. The small generalizability of its results might be considered as a limitation, but the objective was to provide data on this population who is seldom studied in the EM literature. Another limitation in our study was the lack of data on the pursuit of gait training after ICU discharge. In a central RCT on EM in general ICUs [1], the EM protocol was applied throughout the patient hospital pathway. In the current practice of Montpellier University Hospital, BWS walking (or independent walking if feasible) is also trained in the neurological or neurosurgical wards, but this information was not systematically collected for this study.

## Conclusions

This study presented the use in practice of body-weight support walking in ICU for patients admitted to a neurological intensive care unit. Over 78 sessions, it was well tolerated, with 8 session interruptions and only transitory adverse events. It revealed that a suspension device could enable gait training for patients at a stage where neurological impairments would make even bedside standing impossible. It finally illustrated challenges of early mobilization implementation in ICUs in general.

## Data Availability

The datasets used and/or analysed during the current study are available from the corresponding author on reasonable request.

## Abbreviations

BWS: Body weight support
ICU: Intensive care unit
EM: Early mobilization
